# Letter to the Editor: clinical utility of urine DNA for noninvasive detection and minimal residual disease monitoring in urothelial carcinoma

**DOI:** 10.1186/s12943-023-01729-7

**Published:** 2023-02-04

**Authors:** Kaiwei Yang, Hailong Hu, Junlong Wu, Huina Wang, Zhaoxia Guo, Wei Yu, Lin Yao, Feng Ding, Tao Zhou, Wang Wang, Yunkai Wang, Lei Liu, Jing Guo, Shuaipeng Zhu, Xinhao Zhang, Shanbo Cao, Feng Lou, Yuanjie Niu, Dingwei Ye, Zhisong He

**Affiliations:** 1 Department of Urology, Peking University First Hospital, No. 8 Xishiku Dajie, Xicheng District, Beijing, 100034 People’s Republic of China; 2 Department of Urology, The Second Affiliated Hospital of Tianjin Medical University, Tianjin, 300211 People’s Republic of China; 3 Department of Urology, Fudan University Shanghai Cancer Center, Shanghai, 200032 People’s Republic of China; 4 Acornmed Biotechnology Co., Ltd, Beijing, 100176 People’s Republic of China

**Keywords:** Urothelial carcinoma, Urine DNA, utLIFE, Early detection, MRD

## Abstract

Current methods for the early detection and minimal residual disease (MRD) monitoring of urothelial carcinoma (UC) are invasive and/or possess suboptimal sensitivity. We developed an efficient workflow named urine tumor DNA multidimensional bioinformatic predictor (utLIFE). Using UC-specific mutations and large copy number variations, the utLIFE-UC model was developed on a bladder cancer cohort (*n* = 150) and validated in The Cancer Genome Atlas (TCGA) bladder cancer cohort (*n* = 674) and an upper tract urothelial carcinoma (UTUC) cohort (*n* = 22). The utLIFE-UC model could discriminate 92.8% of UCs with 96.0% specificity and was robustly validated in the BLCA_TCGA and UTUC cohorts. Furthermore, compared to cytology, utLIFE-UC improved the sensitivity of bladder cancer detection (*p* < 0.01). In the MRD cohort, utLIFE-UC could distinguish 100% of patients with residual disease, showing superior sensitivity compared to cytology (*p* < 0.01) and fluorescence in situ hybridization (FISH, *p* < 0.05). This study shows that utLIFE-UC can be used to detect UC with high sensitivity and specificity in patients with early-stage cancer or MRD. The utLIFE-UC is a cost-effective, rapid, high-throughput, noninvasive, and promising approach that may reduce the burden of cystoscopy and blind surgery.

**Supplementary Information:**

The online version contains supplementary material available at 10.1186/s12943-023-01729-7.

## Main text

Urothelial carcinoma (UC) is the 12th most common malignancy worldwide, originating from the bladder and upper urinary tract, including the renal pelvis and ureter [1]. Bladder cancer (BC) is the most common urinary tract malignancy [2, 3], and China has a relatively higher percentage of upper tract urothelial carcinoma (UTUC) patients than the Western world [4, 5]. For patients with hematuria, a series of examinations, including computerized tomography (CT)/magnetic resonance imaging (MRI), cystoscopy, urinary cytology, and fluorescence in situ hybridization (FISH), might need to be performed for the diagnosis of UC [6, 7]. Cystoscopy is currently the gold standard for the diagnosis and monitoring of BC [6], but this technique is invasive, uncomfortable, and not suitable for diagnosing UTUC. Of all noninvasive methods for diagnosing UC, urinary cytology has high specificity but relatively low sensitivity [8, 9], while the other 6 assays approved by the Food and Drug Administration (FDA), including NMP22 Bladder Cancer ELISA-Test and NMP22 BladderChek tests (sensitivity 52%–69%, specificity 87%–89%), BTA Stat test (sensitivity 57–82%, specificity 68%–93%), BTA TRAK assay (sensitivity 66%–77%, specificity 5–75%), ImmunoCyt test (sensitivity 60–100%, specificity 75%–84%), and UroVysion test (sensitivity 69%-87, specificity 89–96%), have unsatisfactory sensitivity and/or specificity [9].

Researchers have focused on utilizing urine DNA to develop noninvasive approaches for detecting UC [10–13]. As reported, uCAPP-Seq analysis has confirmed that genetic mutation plays a significant role in the diagnosis and minimal residual disease (MRD) monitoring of UC, with a sensitivity of 83%, specificity of 97%, and area under the curve (AUC) of 0.89 [10]. However, not all UC patients harbor common mutations in genes such as *TERT*, *TP53*, *PIK3CA*, and *ARID1A*. Chromosomal instability has been reported to be nearly ubiquitous in cancer and is a hallmark of human cancer [14]. Chromosomal alterations, including deletions on chromosomes 3, 8, 9, 11, 13, and 17, have been commonly observed in BC and can be measured by karyotyping and FISH [11, 12]. However, assays based on single-dimensional features often yield inadequate detection ability.

Here, we describe a novel noninvasive urine test called utLIFE-UC that addresses the issues described above. The test uses combined assays for genetic alterations and large copy number variants (CNVs) with a customized bioinformatics workflow named the urine tumor DNA multidimensional bioinformatic algorithm (utLIFE). This study aimed to assess the performance of utLIFE-UC for detecting UCs in a multicenter, single-blinded clinical trial.

## Results and discussion

### Study design

A flow diagram summarizing the study design is shown in Fig. S1 (Additional file 1). The algorithm was established in 3 phases: the discovery phase, the training phase, and the validation phase. In the discovery phase, 181 BC tissue samples were used to find candidate markers as described in the supplementary methods (Additional file 2). In the training phase, urine samples from 83 BC patients and 67 healthy controls were used for model construction (Additional file 3: Table S1). In the validation phase, the BLCA_TCGA cohort (281 BC tumor specimens and 393 normal tissue samples) and the UTUC cohort with voided urine samples (11 UTUC patients and 11 nontumor controls) were enrolled in the analysis (Additional file 3: Table S2).

Additionally, we collected urine samples from 31 muscle-invasive bladder cancer (MIBC) patients to monitor MRD (Additional file 3: Table S3). All patients received neoadjuvant therapy (chemotherapy, immunotherapy, or both). Urine samples were collected before neoadjuvant therapy, after 2 cycles of neoadjuvant therapy, and before cystectomy. We did not share the test results with surgeons, and all urine tests were performed before pathology diagnosis.

### Identification of urine tumor DNA markers and their application in the early detection of UC

To define suitable mutation markers for noninvasive diagnostics, the genetic profiles of 181 BC tissues were analyzed to find genes that could cover the maximum number of patients with minimum variants. In all, 155 genes were chosen from the discovery cohort (Additional file 3: Table S4). In the urine cell-free DNA (ucfDNA) from the training cohort, at least one mutation was found in 63.9% of tumor cases, two mutations in 39.6%, and more than two mutations in 8.4%, while no mutation was found in healthy controls (Additional file 1: Fig. S2A). Across the tumor cases, the two most commonly mutated regions were in the *TERT* promoter (47%, 39/83) and *TP53* (18%, 15/83) (Additional file 1: Fig. S2A). Moreover, other genes with a high frequency of mutation in our cohort were *ERBB2*, *ERCC2*, and *FGFR3* (Additional file 1: Fig. S2A). Based on the mutation analysis, a 7:3 training and test cohort was calculated, with the best AUC of 0.819 (63.9% sensitivity at 100% specificity) in the modeling cohort (Additional file 1: Fig. S2B).

Large CNVs were analyzed by using shallow whole-genome sequencing (1 × WGS) data of urine-exfoliated cell DNA (uexDNA). Chromosomal loss and gain were frequently identified in tumor cases but not in healthy controls (Additional file 1: Fig. S2C). For each subsequent sample, we standardized and calculated the CNV score as described in the supplementary methods (Additional file 2). The CNV score was significantly higher in patients than in healthy controls in our cohort (*p* < 0.01) (Additional file 1: Fig. S2D). Then, we tested the CNV score performance according to different segment cutoffs in the modeling cohort with 7:3 training and test sets, and it could discriminate tumor cases from healthy controls with an AUC of 0.934 (Additional file 1: Fig. S2E), sensitivity 86.75 and specificity 97.01% (Additional file 3: Table S5, S6). We also compared the performance between the model with all autosomes and with specific chromosomes from the UroVysion FISH assay. The model used specific chromosomes reached an AUC of 0.864 (sensitivity 79.52, specificity 91.04%) in our cohort, which was inferior to that used all autosomes (*p* < 0.05) (Additional file 3: Table S6).

**Fig. 1:**
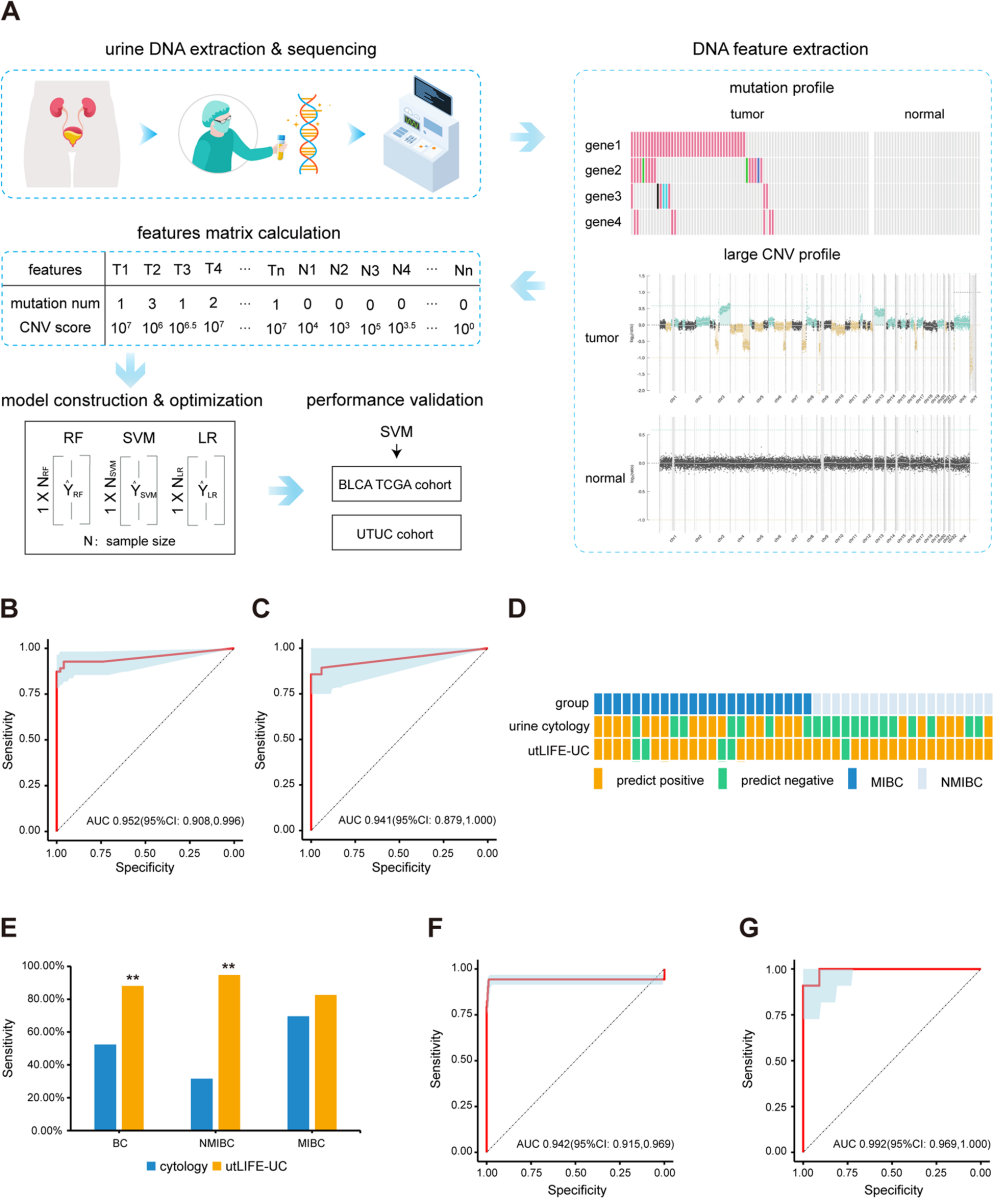
The liquid biopsy testing of urine markers in the early detection of UC. **A** Schematic illustration of utLIFE-UC algorithm. Urine samples were collected from UC patients, as well as healthy controls. The ucfDNA was then extracted from the urine supernatant samples and subject to target sequencing, and the uexDNA was extracted from the urine sediment samples and subject to 1 × WGS. Mutation and large CNV features were extracted, and a base model was constructed. The DNA features were then calculated into a large matrix, which was subsequently trained by three ML methods, RF, LR, and SVM. The SVM method was chosen as the utLIFE-UC algorithm to be validated in independent cohorts. **B** ROC curve of the utLIFE-UC in the training set. **C** ROC curve of the utLIFE-UC in the test set. **D** The landscape of utLIFE-UC and cytology detection results in NMIBC and MIBC. **E** Diagnostic sensitivity of utLIFE-UC compared to cytology in the training cohort (*Fisher’s exact test*; **p < 0.01). **F** ROC curve of the independent TCGA validation cohort. **G** ROC curve of the independent UTUC validation cohort

Next, we constructed the utLIFE-UC model based on a systematic machine learning (ML) framework using combined assays for genetic alterations and large CNVs (Fig. 1A). The detailed procedure used to build the utLIFE-UC model is described below:

1. The feature matrix containing genetic alterations and CNVs was calculated in the modeling cohort and in independent validation cohorts.
2. The modeling cohort was repeatedly randomly split at a 7:3 ratio into training and test sets. In the training phase, three ML methods, namely, random forest (RF), support vector machine (SVM), and logistic regression without regularization (LR), were considered. For the three methods, a 10-fold cross-validation procedure was used to estimate model hyperparameters using only 90% of the training set and the validation of model parameters using the other 10% of the training set. The test set was used to assess the robustness of the algorithm.
3. The independent validation cohorts of BLCA_TCGA and UTUC were used to verify and compare the performance of the three ML methods by AUC, sensitivity, specificity and overall accuracy.

The accuracy of the SVM and LR methods showed superiority compared to the RF method in both the modeling and validation cohorts (Fig. 1B-C, F, Additional file 1: Fig. S3, Additional file 3: Table S7, S8). Additionally, given that the generalization ability of the SVM method is stronger than that of the LR method [15], the SVM method is selected for subsequent research and named as utLIFE-UC model. The overall sensitivity of the utLIFE-UC was 92.78%, with a specificity of 96.00% in the training set (Fig. 1B); in the test set, the sensitivity was 85.71%, with a specificity of 100.00% (Fig. 1C). The two-dimensional model shows better clinical utility than one dimension of mutation or CNV score analysis, demonstrating the advantage of using an ensemble stacked ML model approach with multiple biomarkers. As a measure of relative importance, the proportional contributions to the algorithm score variance were calculated. The large CNV contributed 60%, and the mutation feature contributed 40% in the utLIFE-UC algorithm.

To compare the performance of utLIFE-UC to that of urine cytology for UC diagnosis, 42 non-muscle-invasive bladder cancer (NMIBC) or MIBC patients in the modeling cohort were included for further analysis (Fig. 1D). Overall, the utLIFE-UC assay obtained 2-fold more positive results than cytology (*p* < 0.01) (Fig. 1E). Moreover, utLIFE-UC could detect 82.6% (19/23) of MIBC patients, potentially better than the 69.6% (16/23) detected by urine cytology (*p* > 0.05) (Fig. 1E). Furthermore, in NMIBC patients, the sensitivity of the utLIFE-UC model (94.7%, 18/19) was 3-fold higher than that of cytology (31.6%, 6/19; *p* < 0.01) (Fig. 1E). Our results suggested that the utLIFE-UC model was suitable for both NMIBC and MIBC. Collectively, compared with urine cytology, the utLIFE-UC model seemed to exhibit improved sensitivity, which would be further verified in a larger prospective cohort.

To validate the clinical utility of utLIFE-UC in UC patients with different involved organs or different races, a BLCA_TCGA cohort and a UTUC cohort were used as independent validation cohorts (Additional file 1: Fig. S1). The utLIFE-UC model showed high accuracy in distinguishing BC patients from controls (AUC 0.942, sensitivity 94.31%, specificity 98.73%) (Fig. 1F). The utLIFE-UC score of the BC group derived from the model were distinctively higher than those of the control group (*p* < 0.01) (Additional file 1: FigureS4A). The utLIFE-UC model of the BLCA_TCGA validation cohort showed a NPV of 96.04% (Additional file 3: Table S8), indicating the potential to prevent excessive invasive examination in BC patients. The BLCA_TCGA cohort was chosen for validation because urine DNA from UC patients showed similar regions known to be frequently altered in TCGA BC tumor tissues [16]. The CNV segments were counted from CNV profiles of tissues from the BLCA_TCGA cohort, further suggesting the analysis of TCGA tissue specimens could reflect the classification capacity of the utLIFE-UC algorithm.

Next, UTUC patients and healthy individuals matched for sex and age were enrolled in the UTUC cohort (Additional file 3: Table S2). The utLIFE-UC model could classify UTUCs at a sensitivity of 90.91% and specificity of 90.91% (Fig. 1G), indicating that utLIFE-UC can be potentially aid in diagnostic decisions regarding UTUC. The utLIFE-UC score of UTUC were also distinctly higher than those of the controls (*p* < 0.01) (Additional file 1: Fig. S4B).

These results indicated that the utLIFE-UC model possessed high accuracy and strong clinical utility in the detection of BC and UTUC. It also showed the potential of utLIFE-UC for detection in both Chinese and Caucasian patients. Taken together, our results showed higher sensitivity as well as considerable specificity compared to the six FDA-approved biomarkers [9]. The utLIFE-UC model also showed superior sensitivity (94.31%), which exhibited promising clinical implications in early detection just as uCAPP-Seq (93%) [10] and UroSEEK (83%) [12]. Additionally, due to the informative sequencing data and the extensibility of the ML model, there are opportunities to optimize the clinical utility and implementation of the utLIFE-UC model in a larger prospective cohort.

### Application of the utLIFE algorithm to detect minimal residual disease

**Fig. 2:**
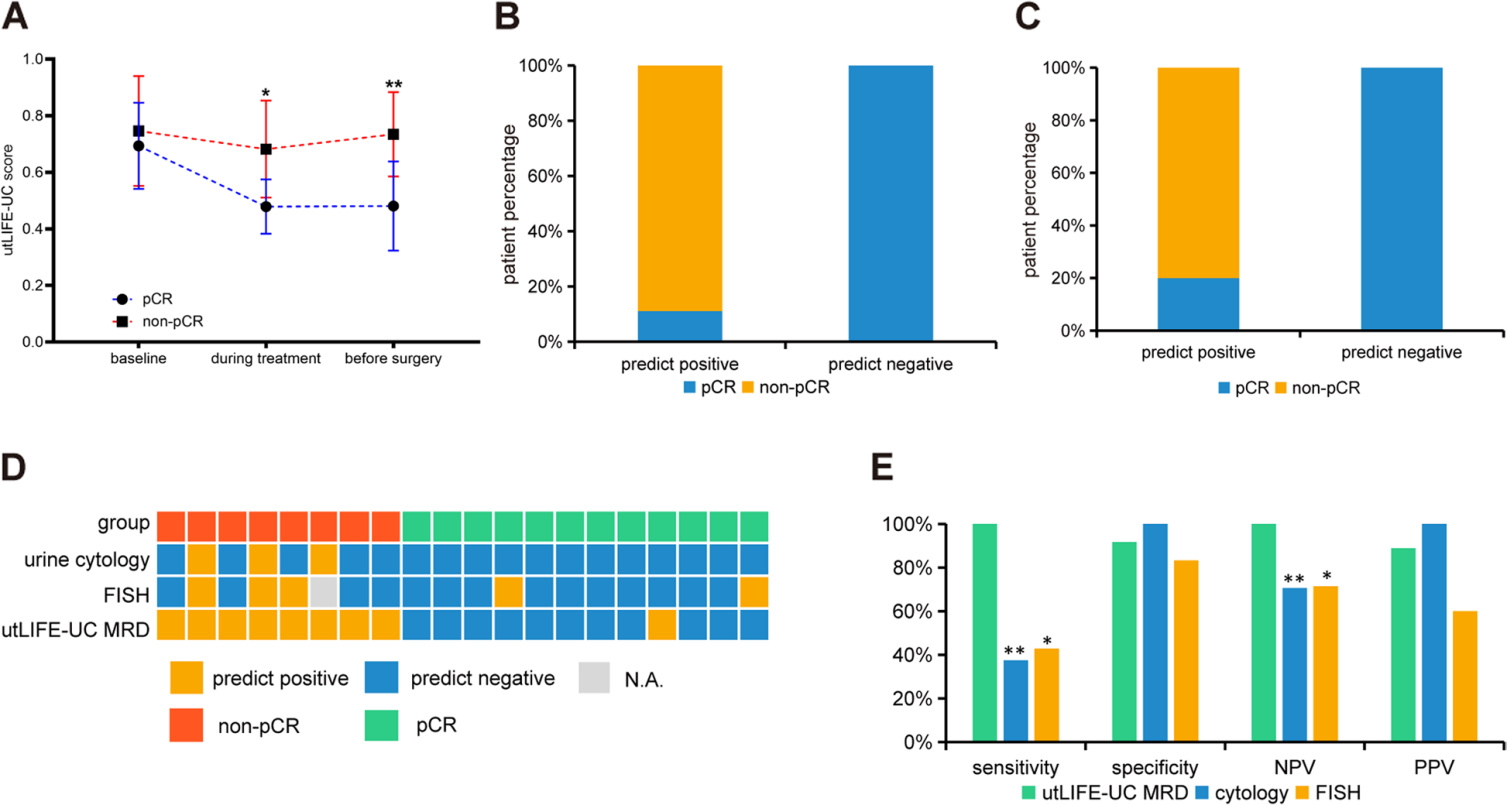
utLIFE-UC MRD analysis in patients with localized bladder cancer. **A** Line chart of the utLIFE-UC score for 2 groups: patients with pCR and patients with non-pCR (student’s *t test*; *p < 0.05, **p < 0.01). **B, C** Stacked bar plots showing the proportions of each group with positive or negative utLIFE-UC scores of the training set (**B**) and the validation set (**C**). **D** The landscape of the utLIFE-UC MRD model, cytology, and FISH. **E** Diagnostic sensitivity and NPV of the utLIFE-UC MRD model compared to cytology or FISH (*Fisher’s exact test*; *p < 0.05, **p < 0.01)

Next, we applied the utLIFE algorithm to the urine samples from patients who underwent radical resection or transurethral resection of bladder tumor (TURBT). The MRD cohort (*n* = 31) was divided into a training set (*n* = 16) and a validation set (*n* = 15) (Additional file 3: Table S3). In the training set, eight patients achieved pathological complete response (pCR), and 8 patients still had residual disease detected in the surgical sample (including partial response (PR) or stable disease (SD), defined as non-pCR). The utLIFE-UC MRD score was similar between pCR and non-pCR urine samples at baseline, while the score was significantly decreased in the pCR group compared to the non-pCR group (*p* < 0.05) during neoadjuvant therapy (Fig. 2A). We constructed an MRD model in the urine samples which collected on the day before surgery, with a sensitivity of 100%, specificity of 87.5%, and negative predictive value (NPV) of 100% (Fig. 2B). In pCR patients, we observed an MRD-negative rate of 75.0% (6/8) at the second time point (during treatment), and 87.5% (7/8) of patients were MRD-negative on the day before surgery, while in non-pCR patients, the MRD-positive rate was 75.0% (6/8) during treatment, and 100% (8/8) of patients were MRD-positive on the day before surgery (Additional file 1: Fig. S5), indicating that the MRD score may represent therapeutic effects in real time. The validation set achieved a sensitivity of 100%, specificity of 80%, and NPV of 100%, and the utLIFE-UC MRD score of the non-pCR group was significantly higher than those of the pCR group (Fig. 2C).

We also compared the utLIFE-UC MRD model to the standard of care. Twenty of 31 patients in the MRD training and validation cohorts underwent urine cytology or FISH assays before surgery (Fig. 2D). The utLIFE-UC MRD model assay was approximately 3-fold more sensitive than cytology (*p* < 0.01) and 2-fold more sensitive than FISH (*p* < 0.05) (Fig. 2E). These results further supported the possibility that utLIFE-UC MRD detection could be a predictor of pathologic response. In conclusion, utLIFE-UC provides potential for accurate noninvasive UC screening prior to the current standard of care in clinical practice.

The MRD cohort study demonstrated a significant correlation between the preoperative utLIFE-UC MRD score and the pathologic response to neoadjuvant treatment. Importantly, pCR was observed in BC patients with utLIFE-UC MRD-negative predictions with an NPV of 100%. Invasive cystoscopy during clinical visits could be avoided in patients with utLIFE-UC MRD-negative predictions due to the high sensitivity and NPV of these predictions. Furthermore, the utLIFE-UC score may reflect the therapeutic evaluation in real time, showing the possibility of advising doctors to choose the right time for surgery. Of note, our MRD detection does not require prior sequencing of tumor tissue. Taken together, utLIFE-UC shows practical clinical utility in early detection and MRD monitoring.

Our study is a case-control study, which does not capture the full spectrum of patients expected to be encountered in the surveillance population. Therefore, more follow-up information needs to be collected in the future to adjust the sensitivity of utLIFE-UC. Additionally, the small size of the UTUC cohort can impact the model performance, likely resulting in an underestimation of sensitivity and specificity. A large, prospective, multicenter cohort study is underway.

## Conclusions

To our knowledge, this multicenter study is by far the largest cohort combining genetic mutations and large CNVs with ML to establish a diagnostic model in UC. The urine-based utLIFE-UC method for profiling mutations and the CNV model demonstrated a clinically feasible test for noninvasive systematic diagnosis and MRD monitoring of UC. The at-home urine self-collection device, which costs approximately the same as cystoscopy, makes the early detection and MRD monitoring of UC more convenient during the era of COVID-19.

## Supplementary Information

**Additional file 1: Supplementary Fig. 1.** Schematic workflow for early detection and minimal residual disease monitoring of UC. **Supplementary Fig. 2.** Urine markers of UC. **Supplementary Fig. 3.** ROC curves of the RF and LR models in the training and test cohorts. **Supplementary Fig. 4.** Point boxplot of the utLIFE-UC score. **Supplementary Fig. 5.** Line chart of utLIFE-UC in continuous samples.

**Additional file 2.** Supplementary Methods.

**Additional file 3: Supplementary Table 1.** Patient characteristics of the training cohort. **Supplementary Table 2.** Characteristics of the UTUC validation cohort. **Supplementary Table 3.** Characteristics of the utLIFE-UC MRD cohort. **Supplementary Table 4.** Mutation used as candidate markers. **Supplementary Table 5.** AUC of different Cutoff_1 value in the training set. **Supplementary Table 6.** The diagnostic performance comparing the CNV score model with all autosomes and with specific chromosomes. **Supplementary Table 7.** Performance of the ML models in modeling cohort. **Supplementary Table 8.** Performance of TCGA validation cohorts by 3 ML models.

## Data Availability

The datasets used and/or analyzed during the current study are available from the corresponding author upon reasonable request.

## Abbreviations

UC: Urothelial carcinoma
UTUC: Upper tract urothelial carcinoma
BC: Bladder cancer
NMIBC: Non-muscle-invasive bladder cancer
MIBC: Muscle-invasive bladder cancer
MRD: Minimal residual disease
FISH: Fluorescence in situ hybridization
CT: Computerized tomography
MRI: Magnetic resonance imaging
utLIFE: Urine tumor DNA multidimensional bioinformatic predictor
TCGA: The Cancer Genome Atlas
FDA: Food and Drug Administration
ROC: Receiver operating characteristic
AUC: Area under the curve
NPV: Negative predictive value
CNVs: Copy number variants
ucfDNA: Urine cell free DNA
uexDNA: Urine exfoliated cell DNA
PKUFH: Peking University First Hospital
FUSCC: Fudan University Shanghai Cancer Center
2^nd^HATMU: The Second Hospital Affiliated to Tianjin Medical University
WGS: Whole-genome sequencing
ML: Machine learning
RF: Random forest
SVM: Support vector machines
LR: Logistic regression without regularization
TURBT: Transurethral resection of bladder tumor
pCR: Pathologic complete response
non-pCR: Including partial response (PR), stable disease (SD)
