# Supplementary material for: Letter to the Editor: clinical utility of urine DNA for noninvasive detection and minimal residual disease monitoring in urothelial carcinoma
Source: Mol Cancer. 2023 Feb 4;22:25. doi: 10.1186/s12943-023-01729-7 (PMC9898696; doi:10.1186/s12943-023-01729-7)
Supplement: Supplementary file 1 — Additional file 1: Supplementary Fig. 1. Schematic workflow for early detection and minimal residual disease monitoring of UC. Supplementary Fig. 2. Urine markers of UC. Supplementary Fig. 3. ROC curves of the RF and LR models in the training and test cohorts. Supplementary Fig. 4. Point boxplot of the utLIFE-UC score. Supplementary Fig. 5. Line chart of utLIFE-UC in continuous samples. [file 12943_2023_1729_MOESM1_ESM.docx]

**Supplemental Figures**

**Table of contents**

Figure S1 2

Figure S2 2

Figure S3 4

Figure S4 4

Figure S5 4


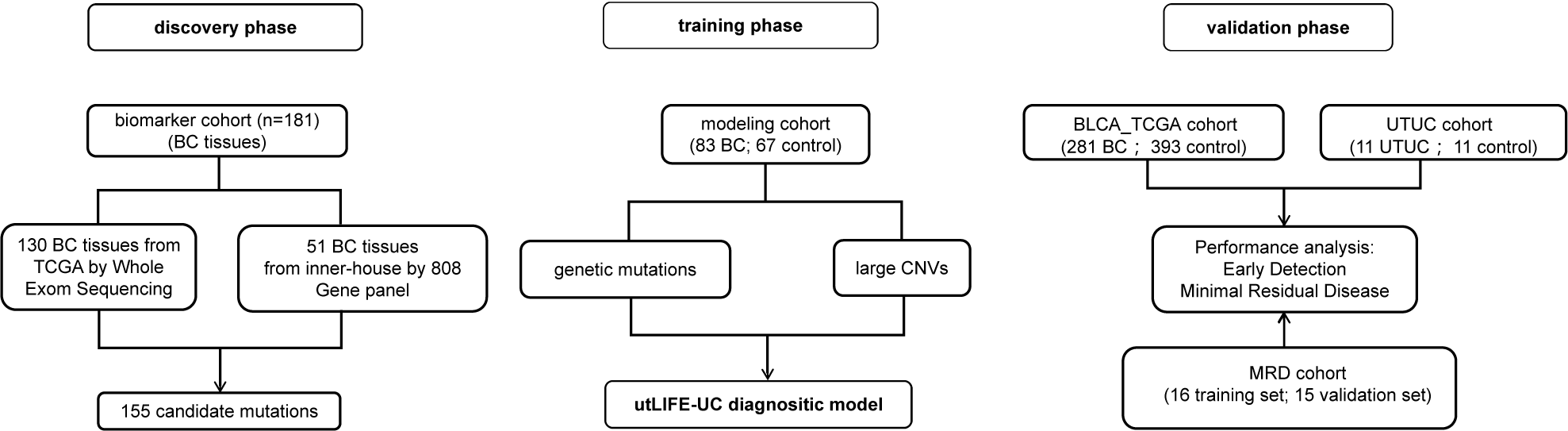


**Figure S1.** **Schematic workflow for early detection and minimal residual disease monitoring of UC.**


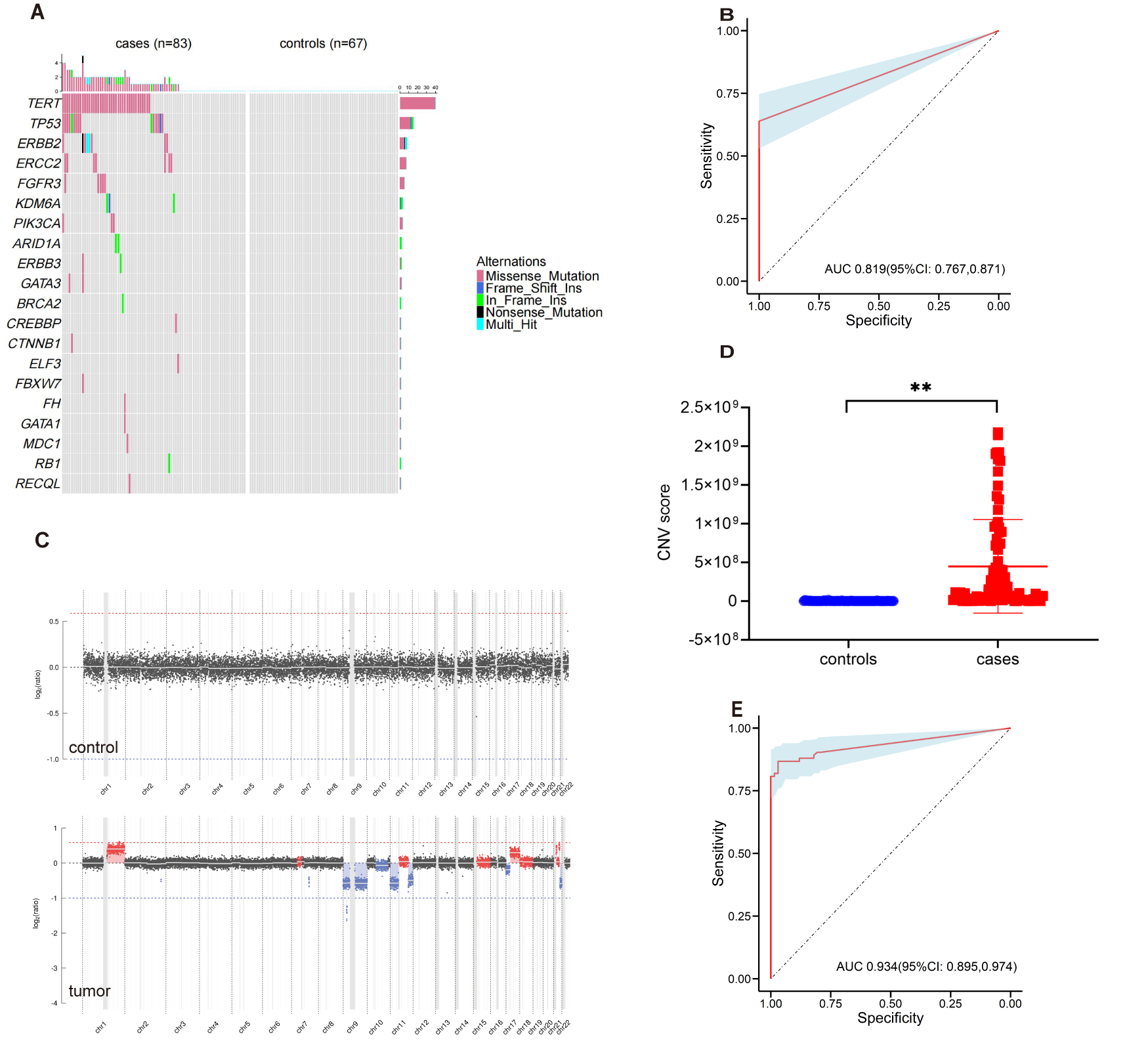


**Figure S2. Urine markers of UC.** (A) Distribution of putative genetic mutations observed across the training cohort in this study. (B) ROC analysis classifies bladder cancer cases versus healthy adults by mutation marker features. (C) Autosome landscapes showed significant differences between tumor cases and healthy controls. (D) Point boxplot of the CNV score in the training cohort (student’s *t-test*; **p<0.01); (E) ROC curve of the modeling cohort by CNV score.


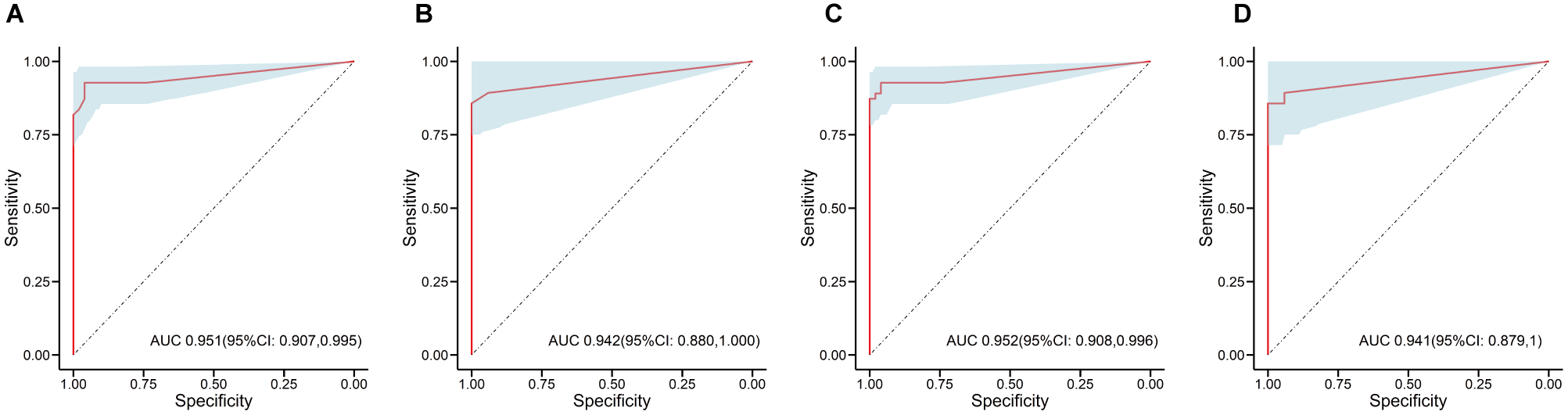


**Figure S3. ROC curves of the RF and LR models in the training and test cohorts.** ROC curves of ML models for bladder cancer cases versus healthy controls in the training (**A,** RF; **C,** LR)/test (**B,** RF; **D,** LR) sets.


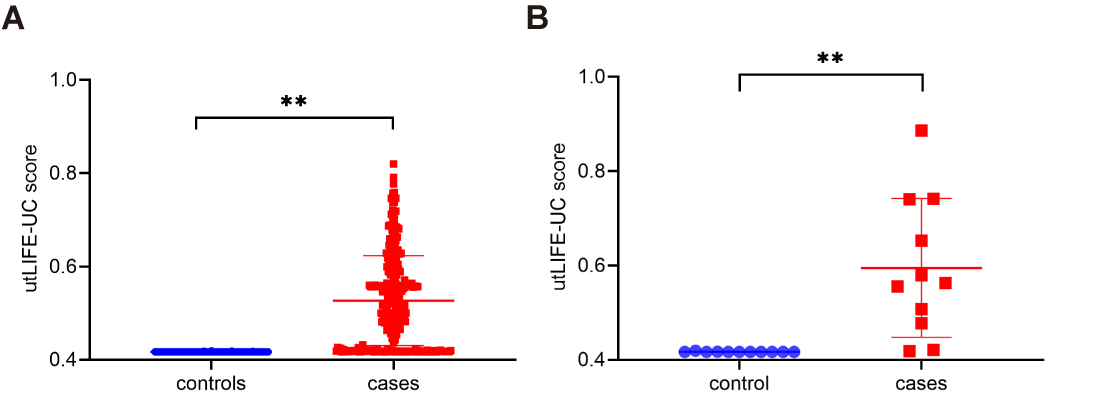


**Figure S4. Point boxplot of the utLIFE-UC score.** (A) The TCGA cohort (student’s *t-test*; ****p<0.01). (B) UTUC cohort (student’s *t-test*; ****p<0.01).

**
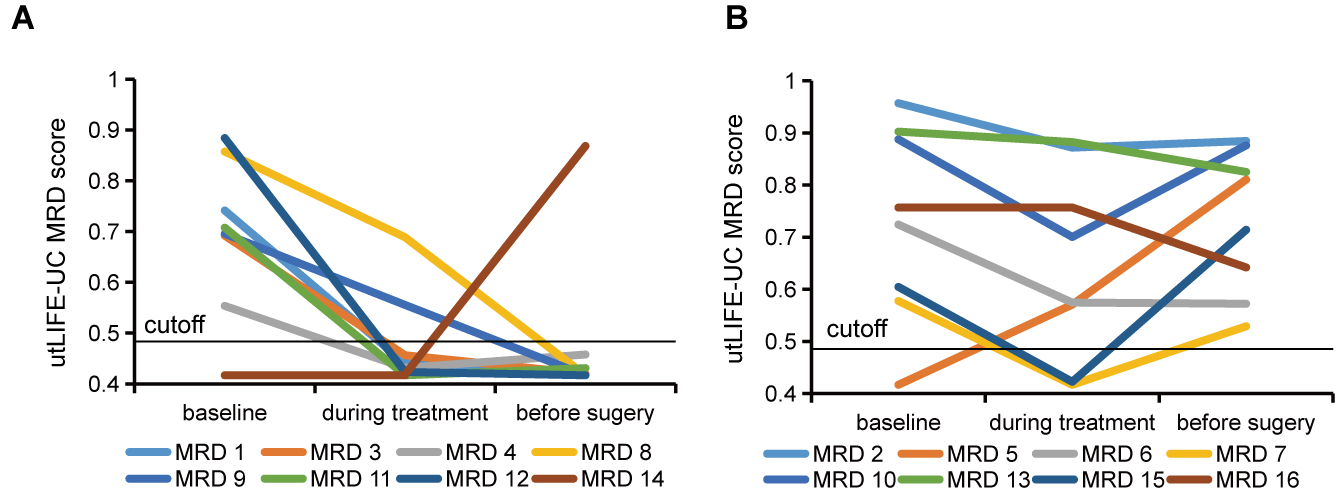
**

**Figure S5. Line chart of utLIFE-UC in continuous samples.** (A) Changes of utLIFE-UC MRD score in the pCR group. (B) Changes of utLIFE-UC MRD score in the non-pCR group.
