# Supplementary material for: Letter to the Editor: clinical utility of urine DNA for noninvasive detection and minimal residual disease monitoring in urothelial carcinoma
Source: Mol Cancer. 2023 Feb 4;22:25. doi: 10.1186/s12943-023-01729-7 (PMC9898696; doi:10.1186/s12943-023-01729-7)
Supplement: Supplementary file 2 — Additional file 2. Supplementary Methods. [file 12943_2023_1729_MOESM2_ESM.docx]

**SUPPLEMENTARY METHODS**

**Study design and sample collection**

In the discovery phase, 130 BLCA_TCGA tissues and 51 BC tissues from The Second Hospital Affiliated to Tianjin Medical University (2^nd^HATMU) were used to find candidate variants. In the training phase, 120 patients who were first diagnosed with BC and 76 apparently healthy controls were recruited from Peking University First Hospital (PKUFH), Fudan University Shanghai Cancer Center (FUSCC) and 2^nd^HATMU between June 2020 and October 2021. After excluding 21 participants with no preoperative urine, 9 samples failed quality control and 16 samples for baseline construction, void urine from 83 BC cases and 67 controls were enrolled in a case-control analysis (Table S1). In the validation phase, genome sequencing data of 281 BC tissues and 393 normal tissues from BLCA_TCGA were used as independent validation cohort 1, and genome sequencing data of urine for 11 UTUC and 11 age-matched and sex-matched healthy controls from PKUFH were used as independent validation cohort 2 (Table S2).

The MRD cohort (31 patients) represented patients with an established diagnosis of BC who were on neoadjuvant tislelizumab, nab-paclitaxel, or both. We also obtained serial urine samples prior to and during neoadjuvant chemotherapy in 15 patients (Table S3).

Morning void urine was collected at the time before any procedures, during patient visits or the day before surgery. Whole blood for germline analysis was collected before any procedures. Fresh-frozen tissue specimens were obtained during diagnostic biopsy. Written informed consent was obtained for samples acquired within PKUFH, FUSCC and 2^nd^HATMU.

**Sample processing**

Urine supernatant was collected using a urine DNA Storage Tube (CWBIO) for urinary cell-free DNA (ucfDNA) extraction. The remaining urine was also collected using a sterile tube containing urine conditioning buffer (UCB, ZYMO) for exfoliated cell DNA (uexDNA) extraction. All the samples were transported to the laboratory at 2°C~8°C within 72 hours. Urine supernatants were centrifuged at 1600 × g and 16000 × g for 10 minutes separately, while urine sediment was centrifuged at 1600 × g for 10 minutes and then stored at -80°C until DNA extraction. Quality control was measured by Micro Drop (BIO-DL) and Qubit 4.0 (Thermo Fisher Scientific) devices. A total of 1-30 ng of ucfDNA and 100 ng of uexDNA were used for library construction and sequenced on Novaseq (Illumina) to generate 150-bp paired-end reads.

**NGS processing**

Quality control statistics and preprocessing of raw sequence data were performed by using fastq (version 0.19.1). Reads were aligned to the hg19 version of the human genome using Burrows–Wheeler Aligner software (BWA, version 0.7.12). PCR duplicates were marked using the MarkDuplicates tool in Picard. IndelRealigner and BaseRecalibrator in the Genome Analysis Toolkit (GATK; version 3.8) were used for realignment and recalibration of the BWA alignment results, respectively. Sentieon TNhaplotyper was used to detect somatic mutations. Softclip type bases and bases with sequencing quality less than 20 were filtered out.

**Candidate marker screening**

To extract suitable mutation markers from urine or tissues, we utilized the same filter criteria and filtered out any mutation also detected in the matched whole blood samples. Mutations with East Asian population frequency >0.001 in GnomAD or ExAC_EAS were also filtered out. The identified markers were further annotated with ANNOVAR for mutation type, associated gene name, location with respect to associated gene, and amino acid change. The mutations were then categorized as silent or nonsilent mutations. Silent mutations included the following: exonic synonymous mutations, intronic mutations, promoter mutations (except the TERT promoter), 5’UTR mutations and 3’UTR mutations. Nonsilent mutations included the following: exonic nonsynonymous mutations, splicing mutations, stop codon mutations, frameshift deletion mutations, frameshift insertion mutations, nonframeshift deletion mutations, and nonframeshift insertion mutations. Silent mutations were filtered out. Nonsilent mutations with mutant allele frequency (MAF) >0.01, reference base depth < 10 and allele base depth <3 were also filtered out. The Cancer Genome Atlas (TCGA) gene list and internal blacklist were also used for filtering.

**CNV biomarker screening**

Quality control statistics and preprocessing of raw data were performed using fastp (version 0.19.1). Reads were aligned to the hg19 version of the human genome using Burrows–Wheeler Aligner software (BWA, RRID:SCR_010910, version 0.7.12). PCR duplicates were marked using Sentieon (version 201911). First, the coverage was calculated for all samples for every 200 k bin. The calculated coverage was corrected by GC context and self-standardization. Sixteen healthy samples were used as controls to eliminate centromeres, telomeres and repeat regions that had no genetic information or considerable noise. All samples were calculated by the following algorithm after standardization:

$$N=\sum segment length(|log2ratio|>Cutoff\_1)$$

If the absolute value of of the segment is greater than Cutoff_1, then the segment is considered to have CNV events. The Cutoff_1 value was obtained through the training set. Cutoff_2 was set to distinguish tumor samples from healthy samples, and its value was also determined by the training set.

**utLIFE-UC algorithm development**

For the utLIFE-UC algorithm, three machine learning models were considered: random forest (RF), support vector machines (SVM), and logistic regression without regularization (LR). For each model type, a combination of variants and CNV_score was evaluated. The modeling cohort (n=150) was randomly split into training and test sets (7:3), and the training set was used to develop the algorithm by a grid search of hyperparameters with 10 k-fold cross-validations, while the test set was used to verify the accuracy and robustness. The SVM model was selected because the SVM outcomes showed superior/comparable performance compared with those of the other 2 models in the validation cohort (Table S5).

**Statistics**

*The results of CNVs and mutations were integrated and evaluated together through three machine learning (ML) algorithms: RF, SVM, and LR.* The sensitivity and specificity of the ML models were estimated by receiver operating characteristic (ROC) curves. The Youden index was used as a summary measure of the ROC curves, and the optimal threshold value (cutoff point) was selected for markers. All statistical analyses were performed by *Fisher’s exact test* or Student’s *t-test* with GraphPad Version 8.0, and a two-sided P value<0.05 was considered significant.
